# External fixator-assisted plating osteosynthesis in a rabbit model of femoral bone defects appears to be a feasible and reproducible surgical technique: preliminary insights from a bone substitute study

**DOI:** 10.1186/s40634-023-00644-6

**Published:** 2023-08-01

**Authors:** F. Vandenbulcke, G. Anzillotti, G. Ravasio, E. Malagoli, P. Conte, B. Balzarini, A. Kirienko, E. Kon

**Affiliations:** 1 Department of Biomedical Sciences, Humanitas University, Via Manzoni 113, Rozzano, 20089 Milan, Italy; 2 Humanitas Clinical and Research Center, External Fixation Unit (Dr Kirienko A), Via Manzoni 56, Rozzano, 20089 Milan, Italy; 3 Department of Veterinary Medicine, University of Milan, Via Festa del Perdono 7, 20122 Milan, Italy; 4 University of Milan, Veterinary Teaching Hospital, Via Dell’Università 6, 26900 Lodi, Italy

**Keywords:** Animal model, Bone regeneration, Non-union, Temporary external-fixator

## Abstract

**Purpose:**

The aims of this study were to assess the complications associated with the use of an external fixator-assisted plate osteosynthesis technique to stabilize a femoral bone defect in a rabbit model and to evaluate if this technique could avoid the mispositioning and the displacement of the femoral fragments during the surgical procedure.

**Methods:**

A preliminary cadaveric animal study was conducted to develop a new technique of external fixator-assisted plating. Thirty rabbits underwent a surgical procedure consisting in the creation of a femoral bone defect and, subsequently an implantation of a bone substitute through the assistance of a temporary external fixator. The fixator’s ability to maintain length and alignment during surgery was documented. All intraoperative complications were prospectively collected.

**Results:**

No complications related to the use of the temporary external fixator were reported. The technique successfully prevented mispositioning and dislocation during plating in all the rabbits.

**Conclusion:**

In a rabbit animal model, the use of an external fixator-assisted plate osteosynthesis technique appears to be feasible and effective in avoiding misposition and rotation of femoral fragments when performing osteotomy and plating to create a mid-diaphyseal femoral defect.

**Supplementary Information:**

The online version contains supplementary material available at 10.1186/s40634-023-00644-6.

## Background

Segmental bone defects can result from malformation, high-energy traumatic events, bone resection due to different conditions such as tumors or infections, or from the treatment of complex non-unions [14]. Their treatment remains a challenge for both patients and surgeons. At the present time, techniques consist in autologous bone graft, allograft, distraction osteogenesis, and bone substitutes [8, 13]. These procedures encompass more than 10% of all skeletal reconstructive surgery cases. Worldwide, 2–3 million grafting procedures are estimated to be performed each year [1]. Recently, huge efforts have been made to develop new materials able to address the treatment of bone defects [2, 12, 15, 18]. Moreover, bone loss is frequently associated with bacterial contamination and subsequent infection [10], therefore it appears fundamental to enrich the treatment options with new materials which also exert an antibacterial activity [7, 9, 11]. Rabbit models appear to be a feasible option to study both innovative materials and implantation techniques [17]. In fact, one of the major difficulties encountered in clinical practice when dealing with such bone defects is the correct maintenance of the limb length and alignment during surgery.

As a part of a study consisting in the evaluation of the effectiveness of bone substitutes, we adopted a rabbit animal model to develop a reliable and reproducible technique to make surgical procedures fast and standardized, with potential benefits for both animal and human studies.

The purposes of this study were:

1. to assess the complications associated with the use of an external fixator-assisted plate osteosynthesis technique to stabilize femoral fragments in a rabbit model of bone defects;
2. to evaluate how this technique may avoid the mispositioning and the displacement of femoral fragments during the surgical procedure.

## Methods

A preliminary cadaveric rabbit study was conducted to evaluate a new technique of external fixator-assisted plating. The in vivo study prospectively included rabbits which underwent a surgical procedure for the creation of a femoral bone defect and filling with a bone substitute (allografts or scaffold). The surgical procedures were carried out by two surgeons (XX, YY) between July 2022 and January 2023.

### Ethics statement

The study was approved by the University of Milan Animal Welfare Organization. The animals were housed in the University of Milan Veterinary Teaching Hospital (Lodi, Italy). The animals were regularly checked by a certified veterinarian responsible for health monitoring and animal wellbeing supervision. All surgical procedures were performed under general anesthesia, and all efforts were made to minimize suffering.

### Animal model

Thirty female New Zealand white rabbits were included in this study. The animals were provided by Charles River Laboratories Italia s.r.l.

### Development of implantation technique

First, an ex-vivo experiment was performed on a rabbit cadaver to become familiar with the implantation technique and to confirm that the implant was able to satisfy the subsequent biomechanical requirements:

- sufficient mechanical stiffness of the plate;
- minimum number of holes for each fragment (at least two holes).

During a second ex-vivo experiment, we introduced the use of a novel external fixator-assisted osteosynthesis in order to:

- easily establish plate positioning before the osteotomy;
- prevent mispositioning and dislocation of proximal and distal fragments during the time elapsed between the osteotomy and the fixation with a plate and screws;
- avoid prolonged surgical time trying to restore normal length and rotation of the femoral fragments.

### Surgical procedures

The rabbits were anesthetized via inhalation of isoflurane (3%; Merial, Italy). All animals received a preoperative intramuscular single injection of cefazolin (30 mg/kg; Cefamezin, Teva, Italy). The left lower limb was shaved and disinfected with 2% chlorhexidine gluconate and 70% isopropyl alcohol solution. The animals were placed on sterile drapes, the bodies were covered with sterile sheets to prepare the surgical field.

A routine lateral approach to the femur was made. Skin and fascia were incised over 6–7 cm in length. The femoral shaft was exposed by blunt dissection of the muscular tissues.

A 10 mm mid diaphyseal femoral defect was then created using an oscillating saw (Stryker, Precision Thin 9.0 × 0.38. 25.0 mm). A bone substitute (allograft or scaffold) was inserted in the defect. The femur was stabilized with a plate and screws. The stability of fixation was manually assessed. The muscular planes were closed with a continuous suture with Vycril 2/0 and the skin with intradermic technique (Vycril Rapid 3/0).

The fixator’s ability to maintain femoral length, alignment and rotation during surgery was documented. All intraoperative complications were prospectively recorded.

### Cadaveric study: choice of the correct implants

**Fig. 1:**
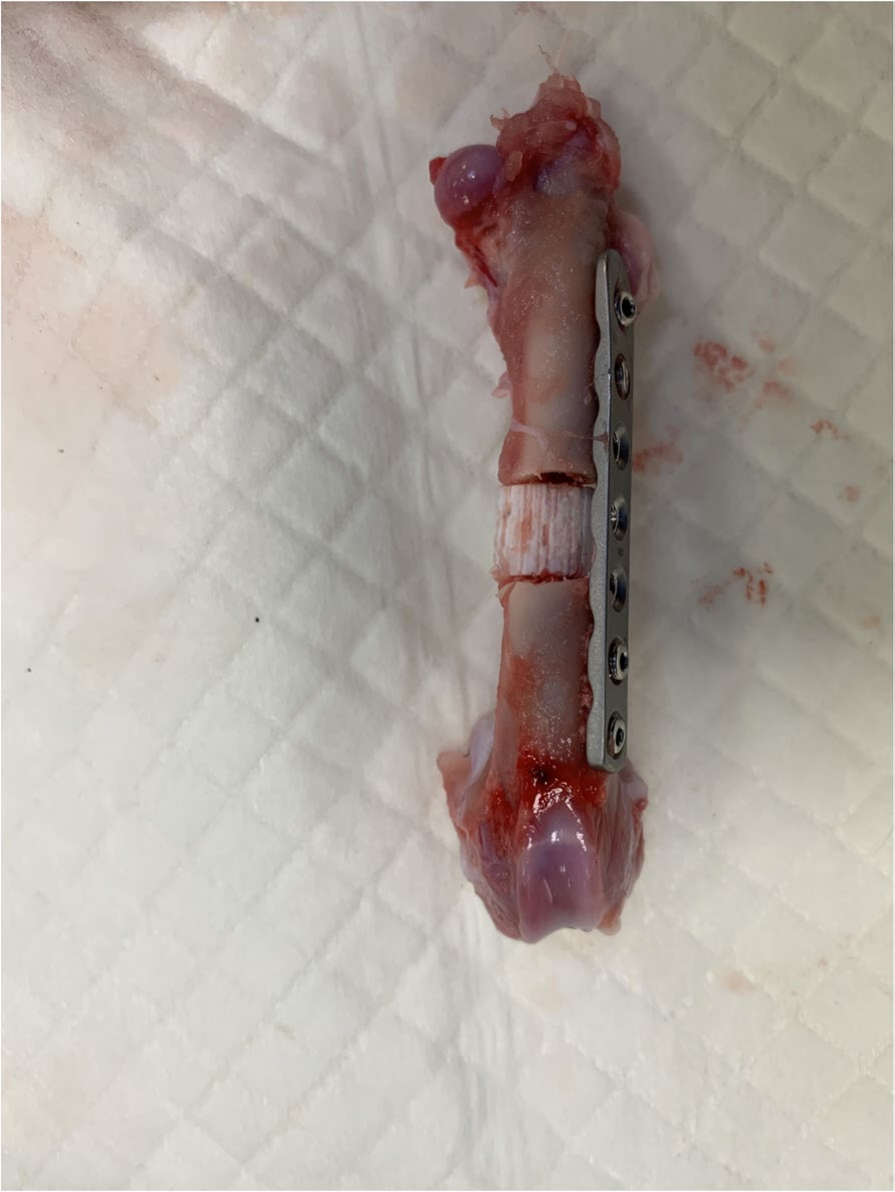
Harvested femur after cadaveric study for the assessment of the most appropriate screw diameter and plate length

During the first ex-vivo experiment conducted on a rabbit cadaver, we chose a Stainless Steel 2.4 mm Locking-Compression Plate (Synthes Vet), 7 holes, 56 mm of length. This was the best compromise between sufficient mechanical stiffness of the plate and minimum number of holes for each fragment (Fig. 1).

### Cadaveric study: technique development

**Fig. 2:**
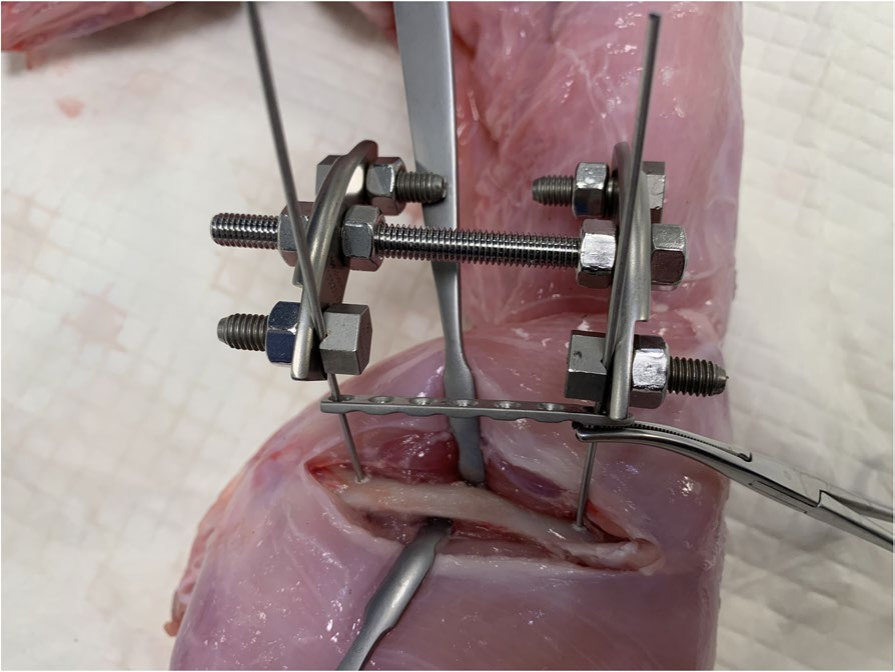
Cadaveric study for development of the new technique

During the second ex-vivo experiment, we carried out a new technique of external fixator-assisted plating in a rabbit model of femoral defect to make the surgery less time consuming and more feasible and reproducible (Fig. 2).

## Results

### Development of the implantation technique

**Fig. 3:**
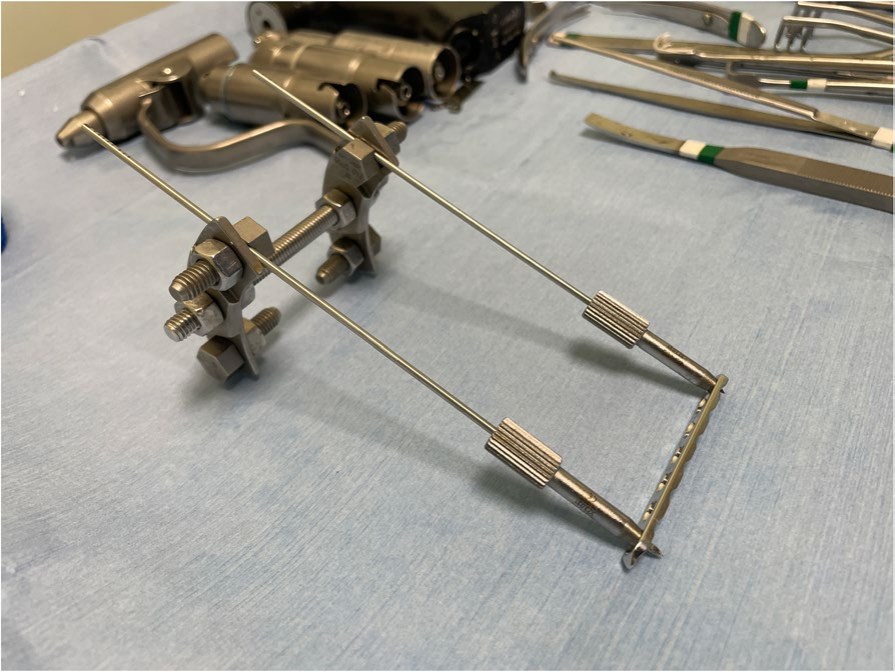
Before skin incision a construct in pre-assembled consisting of the 2.4 mm LCP plate, two threaded drill guides, two 1.8 mm Kirschner wire and the temporary external fixator

**Fig. 4:**
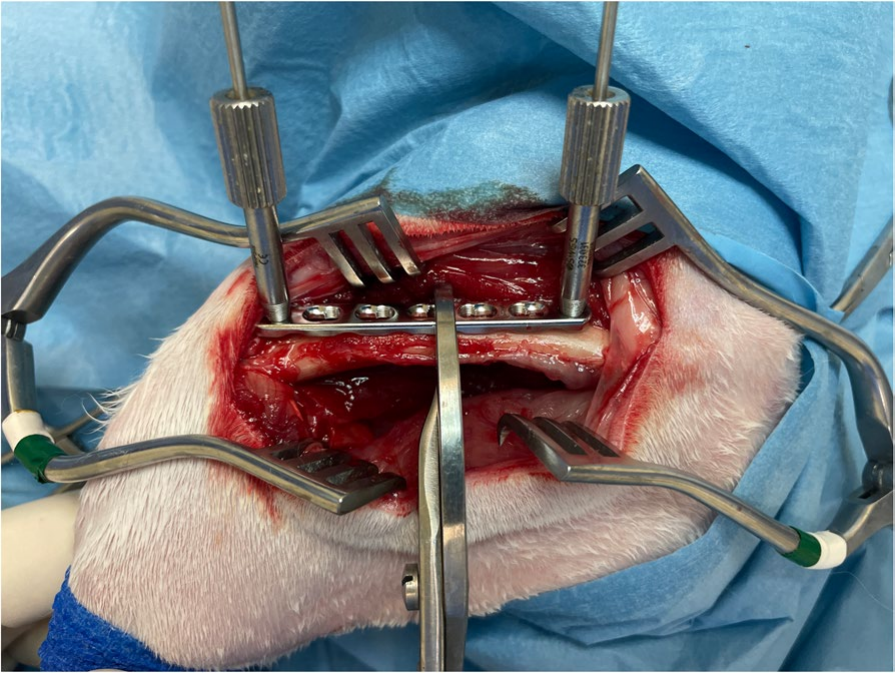
The pre-assembled construct is placed in the desired position and stabilized to the bone with a self-centering bone holding forceps

**Fig. 5:**
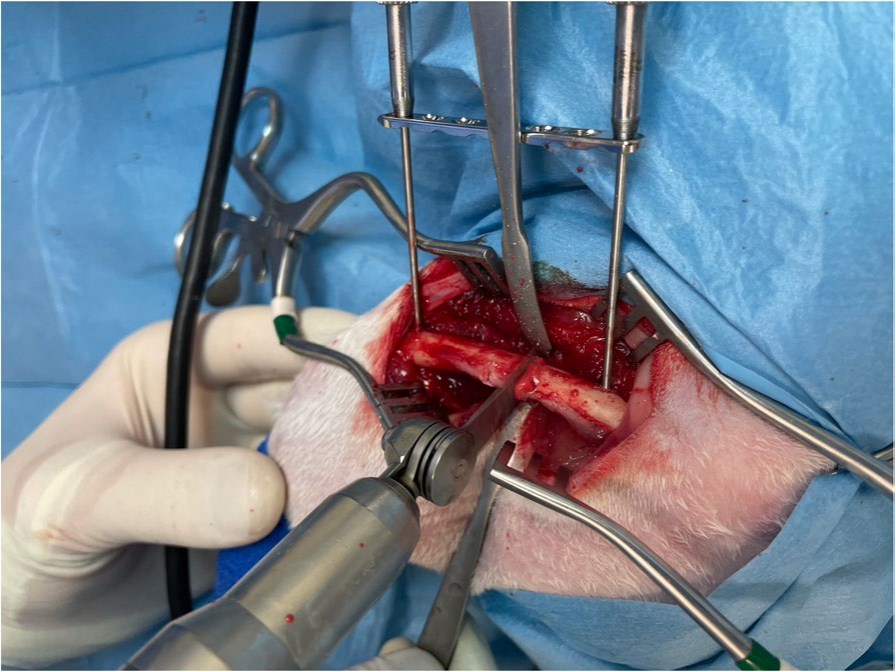
Once the temporary external fixator is fixed to the femur, the plate is freely slid upward along the Kirschner wires. The osteotomies are performed using an oscillating saw

**Fig. 6:**
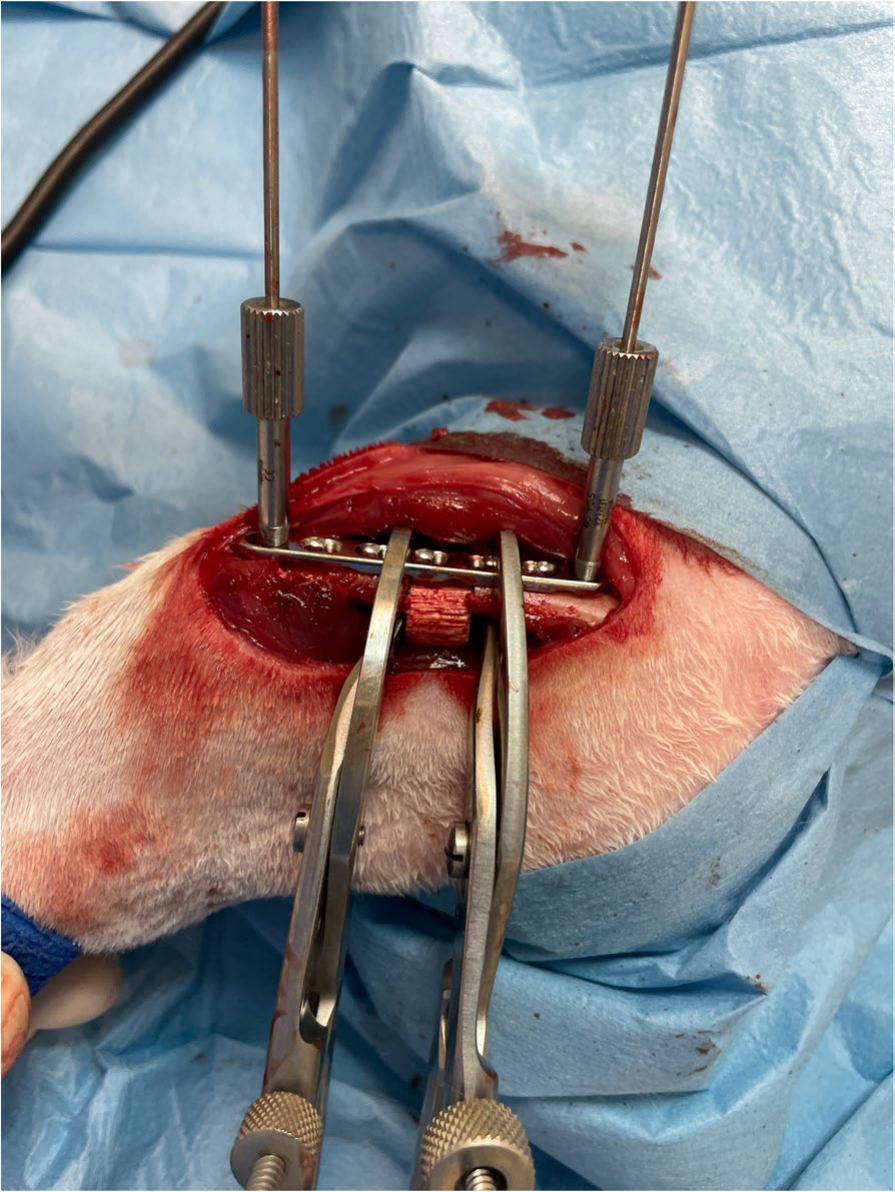
The scaffold is inserted in the defect and the plate is slid downward and stabilized to the bone with two self-centering bone holding forceps

The procedure consisted of the following steps:

- two threaded drill guides were inserted in the two holes at the end of the 2.4 mm LCP plate;
- the temporary external fixator was composed of two curved plates connected by one threaded rod of the same length as the 2.4 mm LCP plate;
- two slotted bolts were placed on the empty holes of the curved plates;
- two more holes were left empty for further wires to increase stability;
- the distance between the two curved plates is adjusted in order for the slotted bolts to be at the same distance as the threaded drill guides;
- two 1.8 mm Kirschner wires (of the same diameter of the drill) were inserted through the threaded drill guides and the slot of the bolts to join the plate and the fixator together, in order to obtain a construct assembled before skin incision (Fig. 3; Video 1);
- after the surgical approach to the femur was performed, the pre-assembled construct was placed in the desired position and stabilized to the bone with a reduction forceps (Fig. 4);
- the two 1.8 mm Kirschner wires were inserted bicortically;
- the nuts were tightened on the slotted bolt to fix the wires to the frame;
- the reduction forceps were removed;
- the plate was freely slid upward along the Kirschner wires (Video 2);
- the bone graft substitute was approached to the mid-diaphysis to confirm the size of resection and to draw the landmark for osteotomies;
- using an oscillating saw, two osteotomies were performed in order to resect 1 cm of femoral diaphysis (Fig. 5);
- the bone graft substitute was inserted in the defect;
- the plate was slid downward and stabilized to the bone with a reduction forceps (Fig. 6);
- two 2.4 locking screws were placed on the second hole proximally and on the sixth hole distally;
- the external fixator, the wires and the drilling guide were then removed;
- two more 2.4 mm locking screws were placed on the first hole proximally and on the 7th hole distally.

### In-vivo study

**Fig. 7:**
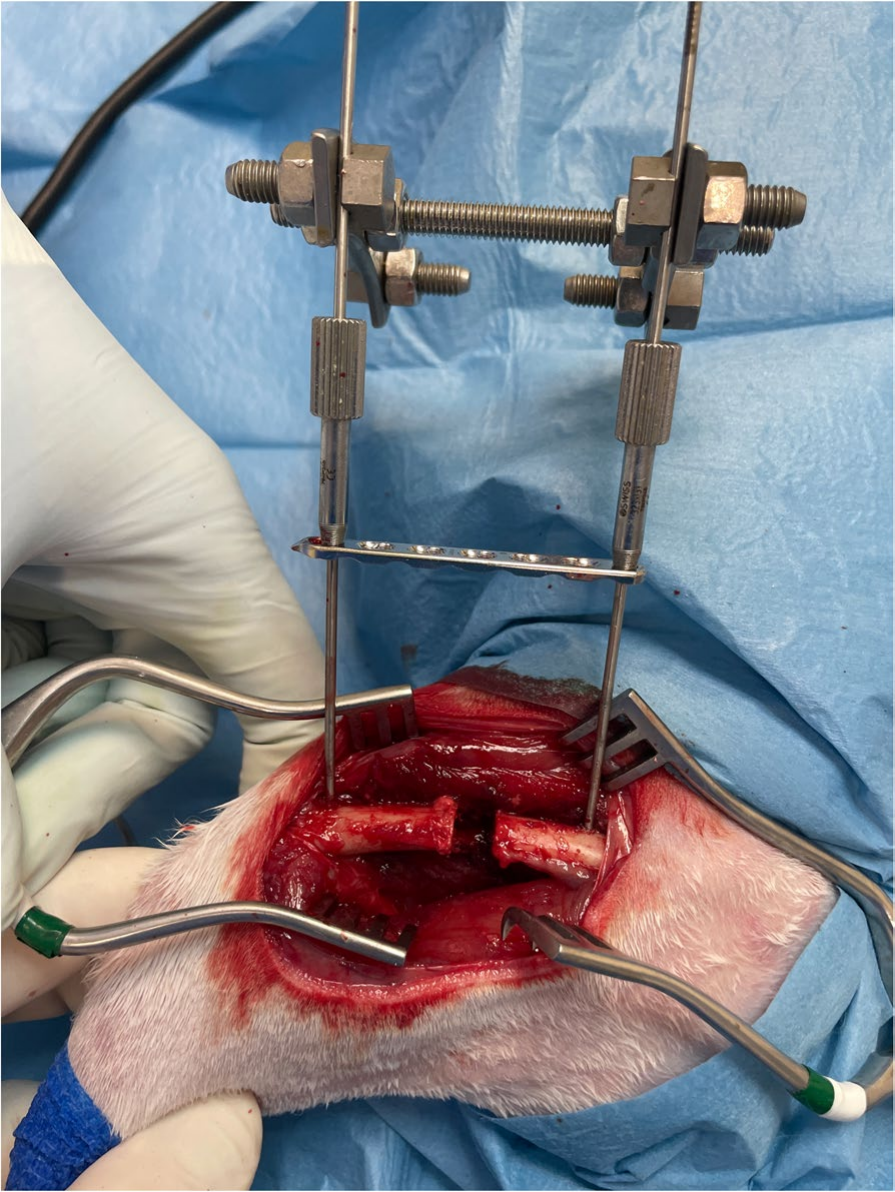
The temporary external fixator prevents shortening and dislocation after resection

Thirty consecutive rabbits were included in the in-vivo study. All rabbits underwent the standardized surgical procedure developed in the ex-vivo animal model. A notable efficacy of the technique developed was observed in avoiding the mispositioning and the displacement of femoral fragments during the surgical procedure. The limitations given by the restricted surgical field of the rabbit femur were overcome by the effective procedure used. No complications were recorded related to the use of the temporary external fixator intraoperatively. No cases of femoral fragments mispositioning or dislocation were observed. The technique was considered successful in preventing shortening and dislocation during plating in all rabbits (Fig. 7). A successful bone substitute implantation was achieved in all the rabbits.

## Discussion

The main finding of this study was the development of a new surgical technique able to successfully prevent mispositioning and dislocation during plating osteosynthesis in a rabbit model of femoral bone defects.

The study confirmed that the technique was feasible in all rabbits. This technique allowed to temporarily stabilize the femur during surgical procedure. The external fixator was placed before the first osteotomy and removed after fixation with plates and screws, thus avoiding shortening and displacement of the fragments. Since two wires were used, each fragment was fixed by only one wire, thus permitting displacement around the axis of the wire. However, we found no significant displacement during surgery. During our cadaveric study, we tried to fix each fragment using two wires placed on different planes, one of which passed in the drill guide and the other was outside the plate in order to improve temporary fixation. We finally decided to abandon this strategy because was time- and space-consuming. However, a little displacement in one axis is easily managed compared to gross shortening and displacement in the three axes of the space.

External fixator-assisted reduction is a well-known technique in traumatology, several authors reported the use of external fixator to restore anatomical alignment and length before internal fixation. Shymon and co-workers [16] described a percutaneous technique that sequentially reduces and aligns distal femur fractures with an anterior external fixator before definitive fixation with a lateral distal femoral locking plate. Gopinathan and colleagues [3] proposed a Fixator Assisted Submuscular plating Technique (FAST), which minimizes the surgical time and simplifies the submuscular plating in pediatric femur fractures. Lee and co-workers [5] described a reproducible MIPO technique that used an external fixator during the procedure as a tool for reduction and maintenance in patients with humeral shaft fracture. Hou’s group [4] employed a circular external fixator to assist in the reduction of calcaneal fractures. For instance, Lee and colleagues [6] suggested the use of a fixator-assisted less invasive plate osteosynthesis technique to stabilize an opening-wedge high tibial osteotomy in the treatment of proximal tibia vara, thus allowing precise correction with fine tuning to accomplish preoperatively planned alignment. However, to our knowledge, no authors have suggested so far the use of such a pre-assembled construct of plate and fixator. Indeed, this technique involves the placement of the plate in the desired position at the beginning of the surgery, immediately after the surgical approach. It is never moved thereafter until definitive fixation is achieved. It is just slid upward along the Kirschner wires in order to perform osteotomies, resect the femoral segment and insert the bone substitutes. Then the plate is slid downward and stabilized to the bone. This construct was not bulky and allowed us to save space, which was mandatory in a such narrow environment. The implantation of the temporary fixator and of the plate at the same time would not have been possible otherwise. However, our study is characterized by some limitations. The lack of a control group operated without temporary external fixator strongly limits our ability to discern if this technique may be defined superior over the implantation of the plate alone in reducing both the mispositioning or rotation of the fragments themselves and in the reduction of surgical time.

## Conclusions

In a rabbit animal model, the use of an external fixator-assisted plate osteosynthesis technique appears to be feasible and effective in avoiding misposition and rotation of femoral fragments when performing osteotomy and plating to create a mid-diaphyseal femoral defect.

## Supplementary Information

**Additional file 1: Video 1.** A pre-assembled construct consisting of the 2.4 mm LCP plate, two threaded drill guides, two 1.8 mm Kirschner wire and the temporary external fixator.

**Additional file 2: Video 2.** The plate slides freely upward and downward along the Kirschner wires.

## Data Availability

Not applicable.
